# Aβ Beyond the AD Pathology: Exploring the Structural Response of Membranes Exposed to Nascent Aβ Peptide

**DOI:** 10.3390/ijms21218295

**Published:** 2020-11-05

**Authors:** Valeria Rondelli, Mario Salmona, Laura Colombo, Giovanna Fragneto, Giulia C. Fadda, Laura Cantu’, Elena Del Favero

**Affiliations:** 1 Department Medical Biotechnologies and Translational Medicine, Università of Milano, Via F.lli Cervi, 93, 20090 Segrate (MI), Italy; valeria.rondelli@unimi.it (V.R.); elena.delfavero@unimi.it (E.D.F.); 2 Department of Molecular Biochemistry and Pharmacology, Istituto di Ricerche Farmacologiche Mario Negri IRCCS, Via Mario Negri, 2, 20156 Milano, Italy; laura.colombo@marionegri.it; 3 Institut Laue-Langevin, 71 Avenue des Martyrs, BP 156, 38000 Grenoble CEDEX, France; fragneto@ill.fr; 4 CSPBAT UMR 7244, UFR SMBH, Université Sorbonne Paris Nord, 74 rue Marcel Cauchin, 93017 Bobigny, France; giulia.fadda@cea.fr; 5 Laboratoire Leon Brillouin, CEA Saclay, F-91191 Gif sur Yvette CEDEX, France

**Keywords:** brain pathology, aging, membrane active peptides, Aβ, lipid rafts, gangliosides, cholesterol, SANS, SAXS, neutron reflectometry

## Abstract

The physiological and pathological roles of nascent amyloid beta (Aβ) monomers are still debated in the literature. Their involvement in the pathological route of Alzheimer’s Disease (AD) is currently considered to be the most relevant, triggered by their aggregation into structured oligomers, a toxic species. Recently, it has been suggested that nascent Aβ, out of the amyloidogenic pathway, plays a physiological and protective role, especially in the brain. In this emerging perspective, the study presented in this paper investigated whether the organization of model membranes is affected by contact with Aβ in the nascent state, as monomers. The outcome is that, notably, the rules of engagement and the resulting structural outcome are dictated by the composition and properties of the membrane, rather than by the Aβ variant. Interestingly, Aβ monomers are observed to favor the tightening of adjacent complex membranes, thereby affecting a basic structural event for cell-cell adhesion and cell motility.

## 1. Introduction

Recent studies have shown that amyloid beta (Aβ) monomers have multiple physiological functions, crucial for brain homeostasis [1,2,3,4]. However, to date, the focus has been on the role they play in the pathological route of Alzheimer’s Disease (AD). In fact, although produced by cleavage as intrinsically disordered peptides [5], they are very prone to acquiring a secondary structure upon binding to a partner [6], thus initiating the production of toxic soluble oligomers that are considered to be principally responsible for the pathology onset and progression of AD [7,8,9]. Therefore, the wealth of related literature, using both in vitro and in vivo experimental models, has addressed the toxic effect displayed by Aβ oligomers [10], with the aim of unravelling their structural properties in relation to their impact on cells. These included the detergent effect, carpeting, and pore formation, that alter the structure of cells and threaten their integrity [11,12], eventually leading to neuronal dysfunction. Moreover, the investigation of the role of cell membranes in the interaction with Aβ has been mainly restricted to their templating influence on the downstream process of amyloid aggregation along the pathological route [13,14,15,16].

Conversely, the role of the monomeric species of Aβ has mainly been neglected. Notably, unlike Aβ oligomers, Aβ_1–40_ and Aβ_1–42_ monomers are non-toxic [17]. Questions regarding the physiological role of Aβ peptide, why and when it is normally produced, and its route and fate outside of its involvement in severe neurodegenerative pathologies, have only recently begun to emerge in the literature. Clinical data have given rise to the hypothesis that it is part of the brain’s mechanism to mitigate or repair neuronal damage or insult, ranging in duration and gravity from short-term sleep deprivation to chronic diseases of the central nervous system [18,19,20]. We wondered whether the nascent Aβ monomer could be involved in these physiological mechanisms. We hypothesized that the nascent Aβ monomer may interact with its membrane environment on its way to its target, because the membrane is the closest site of interaction for the peptide when it has been cleaved from its parent amyloid precursor protein (APP) [1,2,18,19,20]. We then planned to address this latter structural issue using an experimental approach.

The study presented in this paper reports about the structural response of paradigmatic model membranes upon interactions with nascent Aβ monomers with the aim of providing a structural experimental ground beneficial for the investigation of unexplored physiological aspects. While often approached using simulation methods [21], the main hurdle in experimentally addressing the structural, as well as the physiological, role of Aβ monomers in model systems arises from their rapid progression towards oligomers. We could overcome this problem by using a unique experimental strategy involving the depsipeptide precursors that enable obtaining seed-free reproducible solutions of nascent Aβ monomers [22]. Aβ monomers were put in contact with model membranes to check the occurrence and the extent of their embedding within relevant and paradigmatic model lipid bilayers different in composition, structure, and fluidity, ranging from monocomponent phospholipid bilayers to raft mime membranes enriched in cholesterol and GM1 ganglioside [23,24]. In the context of the biophysics and biochemistry of membranes, this scheme addresses some of the main issues: (a) membrane fluidity and propensity to order, (b) the presence of cholesterol or glycosphingolipids, both affecting the membrane properties and known to interact with Aβ peptides and aggregates, and (c) the raft functional platform.

This chosen experimental approach mimics the fate of nascent Aβ monomers immediately after their release from APP, following β-secretases hydrolysis, irrespective of their possible and eventual involvement in amyloid formation. In fact, the adjacent membrane, for proximity and simple diffusion, is the earliest possible site of interaction. An innovative integrated approach comprising small-angle neutron scattering (SANS), differential scanning calorimetry, X-ray scattering, and neutron reflectometry (NR) enabled us to observe the details of the interaction of Aβ monomers with membranes, with no invasivity.

We revealed that the rules of monomer-membrane engagement and the resulting structural effects are dictated by the chemico-physical properties of the membrane rather than by the Aβ peptide variants.

Notably, we also unveiled an unknown structural role of Aβ monomers in inducing tightening between adjacent raft-mime membranes, likely by entanglement. In a living-system perspective, membrane entanglement might result in a reduction in cell motility, a phenomenon that can be accelerated, for example, in the case of brain infections, when the induction of β-secretase can significantly enhance the formation of monomers in an immune response [25]. Thus, we suggest that nascent Aβ monomers may be involved not only in the pathological and amyloidogenic cascade, but also in the physiological process of aging.

## 2. Results and Discussion

### 2.1. Systems and Experimental Conditions

Paradigmatic model membranes of different compositions were built, ranging from simple phosphatidylcholine (PC)-phospholipid bilayers to complex biomimetic membranes, suitable for investigating whether a direct interaction with nascent Aβ monomers occurred and, if so, the structural effects induced in the various membranes by the presence of the peptide. The choice of model membranes addresses some of their main characteristics: (a) membrane fluidity and propensity to order, (b) the presence of cholesterol or glycosphingolipids, both affecting the membrane properties and known to interact with Aβ peptides and aggregates, and (c) the raft functional platform.

The concentration of the peptide was kept very low, 20 μM, to preserve the monomeric state throughout the incubation period and the experiment, as proven by atomic force microscopy (AFM) (Figure S1a,b) and laser light scattering (Figures S1c,d and S2). Results show that the monomeric state for both species was preserved over longer times than required for the experiments, then, largely within the limits of reliability.

Moreover, the Aβ monomer-to-lipid mole ratio was very low, 0.2% mol/mol, approaching a biomimetic ratio. In some cases, this resulted in the issue of visibility that could be addressed and overcome by the experimental opportunity offered by the neutron spectroscopy techniques. In fact, appropriate H-D isotopic substitution enables reducing or modulating the contrast of membranes to the solvent, while essentially preserving their chemico-physical properties. In the following sections, we describe the structural response of membranes with increasing complexity to the exposure to Aβ monomers.

### 2.2. Single-Component PC-Phospholipid Bilayers

**Figure 1:**
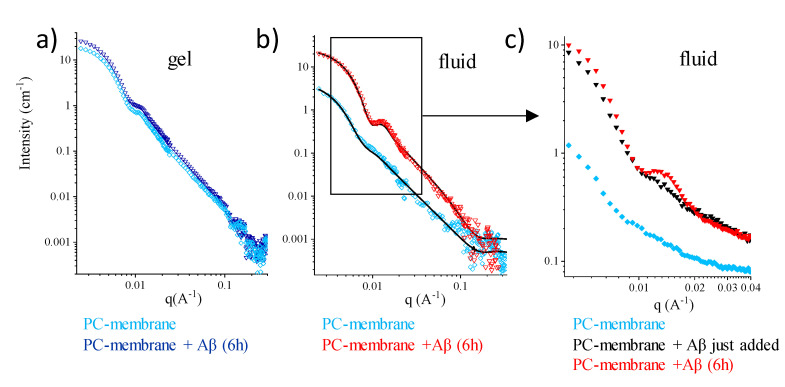
Phosphatidylcholine (PC)-phospholipid bilayers upon interaction with Aβmon (small-angle neutron scattering (SANS)). (**a**) T = 25 °C (gel). Spectra of Dipalmitoyl phosphatydilcholine (DPPC_d75_) unilamellar vesicles in D_2_O (c = 15 mg/mL) before (cyan open diamonds) and after 6 h of incubation with Aβ_mon(1__–__40)_ (blue triangles). (**b**) T = 50 °C (fluid). Spectra of DPPC_d75_ unilamellar vesicles in D_2_O before (cyan open diamonds) and after 6 h of incubation with Aβ_mon(1–40)_ (red triangles). DPPC_d75_ scattered intensity is very low above the chain melting temperature (Tm = 39 °C), as the system is close to the contrast-matching condition. Lines are the fitting curves. (**c**) T = 50 °C (fluid). Focus on the restricted area enclosed in the rectangle of panel (**b**) added with the spectrum of the DPPC_d75_ unilamellar vesicles in D_2_O just after injection of the solution containing Aβ_mon(1–40)_.

The basic PC-phospholipid bilayer allows to clearly address whether membrane fluidity and surface compactness interfere with Aβ monomers admission. In fact, the structural properties of PC-phospholipid bilayers are well known both in the gel and in the fluid phases, as well as their order-disorder transition, occurring as temperature is raised in a range typical of the PC-phospholipid acyl chains [26]. Closed PC-phospholipid unilamellar vesicles (LUVs) (~80 nm in size) in D_2_O were observed by SANS (Figure 1). A small amount of freshly prepared nascent Aβ_(1–40)_ peptide (see the Section 3) was then added directly into the measuring cell at a peptide/lipid mole ratio of 0.2% and at a peptide concentration of 20 μM in the final solution. At this concentration, the monomeric peptides free in solution are not visible.

The SANS spectra are shown in Figure 1. After 6 h of incubation onto the gel-phase membrane, the intensity profile was unchanged (cyan diamonds and blue triangles in Figure 1a). The mere upshift in intensity following Aβ_mon_ addition is due to the change in the scattering length density (SLD) of the solvent, and it corresponds to the small amount of H_2_O (20 µL in 250 µL) that was unavoidably added with Aβ_mon_ (the SLD of the solvent decreases from 6.4 × 10^−6^ Å^−2^ to 5.9 × 10^−6^ Å^−2^).

The results of the same experiment performed on the fluid membrane (above the lipid chain transition temperature T_m_) are shown in Figure 1b. At T = 50 °C, the scattering intensity of the pure PC fluid membrane was very low, due to its low contrast against the D_2_O solvent above T_m_. In this case, the observed modification of the spectrum following Aβ_mon_ incubation cannot be simply ascribed to the change in the SLD of the solvent. This is shown in detail in Figure 1c, which focuses on a narrower q-range of significance. Here, the sudden effect detected upon the injection of the 20 µL (in 250 µL) of the Aβ_mon_-H_2_O solution, due to the change in solvent contrast, is followed by membrane remodeling, which is different from what is seen in Figure 1a. Equilibrium is attained after a few hours. Fitting to a closed bilayer model (details in the Section 3) shows that the fluid membrane was mainly affected in the outer leaflet (SLD decreases from 5 × 10^−6^ Å^−2^ to 4.3 × 10^−6^ Å^−2^; the structural parameters are reported in Table S1). The data show that, above T_m,_ interaction occurs between Aβ_mon_ and the membrane surface. A reduction in the polydispersity of the LUVs size (from 0.28 to 0.23) suggests that a spontaneous curvature was likely conferred to the bilayer.

Similar results were obtained for the PC vesicles in interaction with Aβ_mon(1–42)_. Dimyristoyl phosphatydilcholine (DMPC_d67_, T_m_ = 19 °C) LUVs were observed at T = 25 °C, in their fluid phase (Figure S3a). Results show that interaction occurs between the fluid PC membrane and Aβ_mon(1–42)_, inducing a membrane remodeling in 6 h (Figure S3b and Table S1). Moreover, the addition of 20 µL H_2_O in the 250 µL D_2_O lipid solution was tested on the PC LUVs to exclude changes in the intensity profile upon addition of the pure hydrogenated solvent above the chain transition temperature (Figure S4). Interestingly, once remodeling of the fluid membrane occurred due to the exposure to Aβ monomers, it was maintained across the fluid-to-gel transition, as assessed on Dipalmitoyl phosphatydilcholine (DPPC) by the decrease in temperature from 50 °C to 25 °C (Figure S5).

Overall, the SANS results for single-component PC LUVs indicate that the membrane must display some features typical of the fluid-phase structure in order to interact with nascent Aβ. Moreover, even in a small amount, Aβ monomers affect the surface of the membrane, increasing its interface-to-volume ratio. Notably, this occurs for both Aβ molecular species.

**Figure 2:**
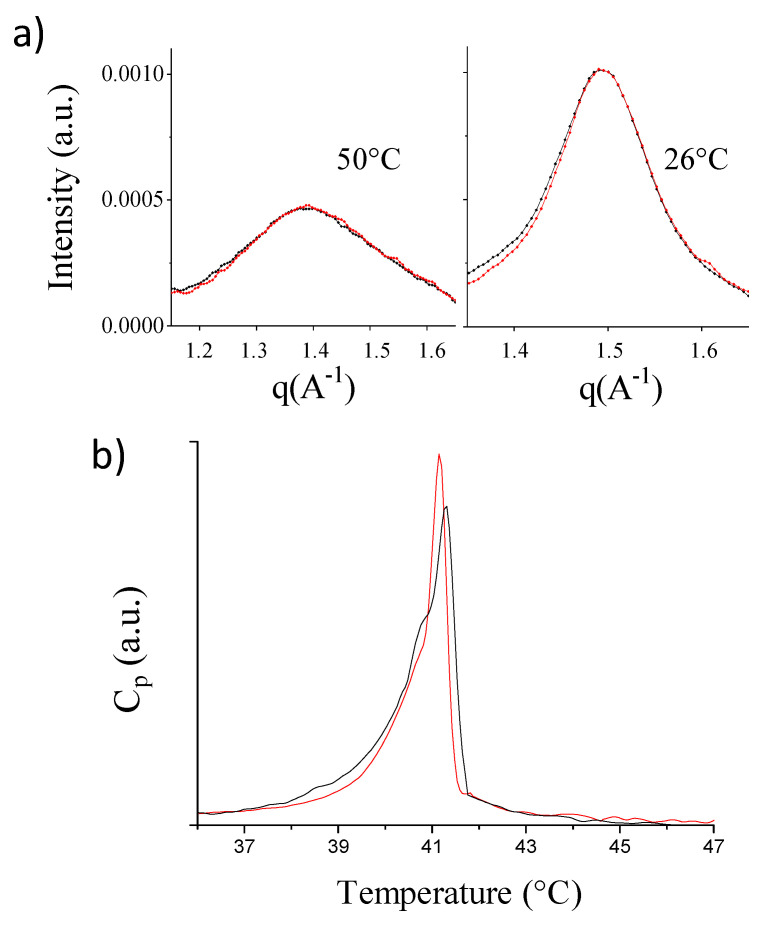
PC-phospholipid bilayers upon interaction with Aβ_mon_ (wide-angle X-ray scattering (WAXS), differential scanning calorimetry (DSC)). (**a**) Wide Angle X-ray scattering (WAXS) of DPPC vesicles before (black curves) and after 1 h contact at T = 50 °C with Aβ_mon(1–42)_ (red curves) measured at T = 50 °C (left panel) in the fluid phase of lipid chains and at T = 26 °C (right panel) in the gel phase. (**b**) Differential scanning calorimetry (DSC). Upward T-scan of DPPC vesicles before (black line) and after contact with Aβ_mon(1–42)_ (red line) showing the downshift of the main peak from 41.3 to 41.15 °C (shoulder from 40.8 to 40.7 °C). c = 20 mg/mL, scan rate 0.25 °C/min.

More insight into the nature and depth of this structural response can be obtained using focused experimental techniques. Wide-angle X-ray scattering (WAXS) experiments were used to inspect membranes on the length-scale of the lipid chains distance. The WAXS spectra reported in black in Figure 2a arise from the naked DPPC vesicles, above (left, (q_structurepeak_ = 1.389 Å^−1^) and below T_m_ (right, q_braggpeak_ = 1.494 Å^−1^, characteristic distance = 4.2 Å). Incubation of Aβ_mon(1–42)_ at T = 50 °C resulted in the spectra reported in red in Figure 2a, above and below T_m_. The WAXS peaks are superimposed, in both the fluid and gel phases, thus indicating that the local arrangement of the lipid chains was not affected by Aβ_mon_ incubation. This result is not surprising, as the Aβ_mon_:lipid mole fraction was very low (DPPC concentration = 20 mg/mL; Aβ_mon(1–42)_: 0.2% mole fraction to lipids, about 1% *w/w*).

Parallel small-angle X-ray scattering (SAXS) experiments (Figure S6) show that the structure of the vesicles is slightly modified upon incubation with Aβ_mon(1–42)_ at T = 50 °C, in the low-q region, which is in agreement with the SANS results.

Overall, the scattering experiments reveal that, while interacting with the surface of the phospholipid fluid membranes, Aβ monomers do not induce a significative modification in the local organization of the lipid core, either in the fluid phase or the gel phase. However, some effect is not unexpected due to the coupling between the surface and the core packing of the lipids, which could affect the propensity of the core to the disordered (or ordered) arrangement. We then investigated the LUVs using differential scanning calorimetry (DSC), which is widely applied to assess the thermotropic properties of phospholipid extruded vesicles with different compositions [27] and to study the interaction of model membranes with active peptides and amyloids [28]. The excess heat capacity profile (Figure 2b) shows the expected peak associated with the main transition, with a shoulder on the left side, which was already observed in the DPPC LUVs at low T-scan rates [27]. The exposure to Aβ_mon(1–42)_ at a peptide:lipid mole ratio of 0.2% induced a ~0.1 °C downshift of the T_m_, with both a ~6% reduction in the transition enthalpy and a ~25% reduction in the peak width. A similar effect was observed for the DMPC closed membranes in the presence of Aβ_mon(1–40)_ (Figure S7).

The DSC results are compatible with the notion that the presence of the peptide on the PC membrane surface may perturb the lateral arrangement of the headgroups without affecting the lateral organization of the chains in both the gel and fluid phases (as evidenced by WAXS). Rather, the transition to the disordered phase is favored by the weaving of Aβ monomers within the phospholipid headgroups layer.

We conclude that, in the case of the paradigmatic pure-PC membranes, the extent of Aβ_mon_-membrane interaction is mainly modulated by the fluidity of the bilayer. Aβ monomers stably adhere to the external surface of PC membranes in their fluid state, where they may benefit from the looser packing of the headgroups to insinuate themselves. They affect the region of the headgroups by increasing the interfacial area per lipid, and they enhance the propensity of the heads to assume the hexatic arrangement rather than the hexagonal surface motif.

### 2.3. Multicomponent PC-Phospholipid-Based Bilayers

The occurrence and structural outcome of the interaction of Aβ with lipid membranes can be modulated by the presence of components that shape the surface that is exposed to the approaching molecules. Several studies have reported on the role played by the presence of gangliosides, and in particular GM1, in the aggregation process of Aβ, promoting the formation of peculiar fibrillar species [14,15]. These studies focused on the conformational changes induced in Aβ monomers by GM1 molecules, which then promotes the membrane-driven aggregation process of the peptides, distinct from the free-standing situation, also as a function of the Aβ/GM1 ratio [16]. In our study, we focused on the interaction between nascent Aβ and the membrane before a peculiar fibrillation process occurs, in particular, on the effect induced by a few Aβ monomers on the structure of the nearby membrane.

We recently reported on the diverse structural effects that Aβ peptides, monomers, and oligomers induce in paradigmatic model membranes containing both GM1 and cholesterol [29]. Such model membranes mimic complex microdomains with prominent biological significance as functional platforms, broadly called rafts. Nonetheless, that previous study did not clarify whether the simultaneous presence of the two compounds is required for the occurrence and modality of membrane reshaping. In has been reported that cholesterol facilitates the interaction of Aβ with GM1 [30]. To obtain some insight, in the present study we also investigated the structural effect that contact with Aβ monomers produces in PC-based membranes containing either GM1 or cholesterol, in the same amount as in the rafts.

#### 2.3.1. Mixed PC-Phospholipid /GM1-Ganglioside Bilayers

**Figure 3:**
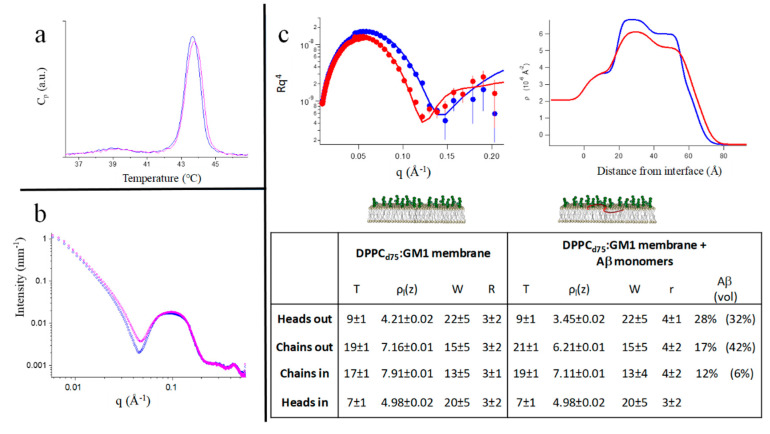
Mixed PC-phospholipid: GM1-ganglioside bilayers upon interaction with Aβ monomers. (**a**) DSC results. Heating scan of DPPC:GM1 vesicles before (blue line, pretransition at T_p_ = 39 °C, T_m_ = 43.65 °C) and after contact with Aβ_mon(1–42)_ (magenta line, T_m_ = 43.75 °C). T-scan rate: 0.25 °C/min. (**b**) SAXS results. SAXS spectra of DPPC:GM1 vesicles before (blue diamonds) and after 1 h incubation with Aβ_mon(1–42)_ at T = 50 °C (magenta open dots). Both spectra were taken at T = 26 °C, gel-phase. (**c**) neutron reflectometry (NR) results. NR spectra (left panel) of the single deposited DPPC_d75_:GM1 membrane, before (blue symbols) and after (red symbols) interaction with Aβ_mon(1–40)_, together with the corresponding contrast fits. In the right panel, the obtained scattering length density profiles. T = 22 °C. Table: Layers parameters, as deduced by the fits of NR spectra, of a single deposited DPPC_d75_:GM1 membrane, before (left section) and after (right section) interaction with Aβ_mon(1–40)_. Layers thickness (Å), scattering length density (SLD) (10^−6^ Å^−2^), solvent penetration (volume %), roughness of interfaces (Å). In the last column, the estimated peptide penetration at different depths within the membrane; in parentheses, the estimated peptide penetration at different depths within a raft-mime membrane DPPC_d75_:Chol:GM1 is reported for comparison, according to Reference [29].

We applied the DSC, SAXS, WAXS, and NR techniques on mixed DPPC:GM1 membranes. DPPC:GM1 LUVs form spontaneously. The DSC profile of the DPPC:GM1 (10:1 mole ratio, total lipid concentration: 2% *w/w* in H_2_O) vesicles is shown in Figure 3a (blue line). The main transition peak (>43 °C) is well above that of the pure DPPC vesicles (~41 °C), as seen in Figure 2b. In the LUVs, the presence of GM1 also broadens the transition peak. Then, the core of the DPPC:GM1 vesicles is more prone to the ordered phase. Less expected is the change induced upon interaction with Aβ_mon(1–42)_ (Figure 3a, magenta line), resulting in a further increase in the chain-melting temperature of the bilayer.

The SAXS spectra of the two systems are shown in Figure 3b. The effect generated by incubation of Aβ_mon_ (1% *w/w*) is compatible with the surface decoration of the vesicles [31], suggesting that the Aβ peptides adhered to the external surface of the DPPC:GM1 vesicles. In the WAXS region, the spectra corresponding to the naked vesicles and the peptide-decorated vesicles were superimposed. Then, the same local order of lipid chains occurs in the two systems, both in the fluid and gel phases, probably mainly dominated by the presence of gangliosides, as also suggested by DSC. The measured further increase of the transition temperature following peptide incubation revealed that, unlike what occurred for the pure PC membranes, Aβ_mon_ did not act as simple spacers in the core or within the headgroups of the target surface. Rather, Aβ monomers enhance the propensity of the DPPC:GM1 bilayers to form ordered phases. We can hypothesize that nascent Aβ, besides being recruited by the membrane, promotes some coordination of the GM1 headgroups on the membrane surface. In addition, molecular dynamics simulation detected hydrogen bonds between Aβ and the sialic acid (Neu5Ac) of the GM1 headgroup, which is likely the preferential site for this interaction [21].

The DSC and X-ray scattering data indicate that a peculiar interaction occurs between Aβ_mon_ and the ganglioside-containing membrane, and the outcome is different from the interaction between Aβ_mon_ and the ganglioside-free membranes. The interaction may be limited to the sugar-containing surface, but the results suggest the possibility of a deeper insertion.

To clarify this point, i.e., the extent of Aβ_mon_ intrusion within the PC:GM1 membrane, we applied NR [32] to single-supported membranes. A supported asymmetric single macroscopic DPPC_d75_:GM1 membrane was prepared (5% total GM1 molar ratio to lipids, asymmetrically residing only in the external leaflet, as described in the Materials and Methods section), and we exposed it to an interaction with Aβ_mon_ dissolved in the contact solvent, at T = 22 °C. In this experiment, the lipids-to-Aβ_mon_ mole ratio was definitely lower than it was in the bulk experiments (peptide:lipid = 1:3 mole ratio), while the peptide concentration in the solution in contact with the single deposited membrane was in the order of micromolars, as seen throughout this study, approaching biomimetic conditions.

The NR spectra are reported in Figure 3c together with the corresponding fits (left panel) and the obtained SLD profiles (right panel). The structural parameters are reported in the table presented in Figure 3c. The estimated peptide penetration results showed that Aβ_mon_ was able to penetrate the membrane, reaching the inner chains layer. Aβ_mon_ also produced an increase in the hydrophobic thickness of the PC:GM1 membrane (~10%), likely stretching the hydrophobic chains, an effect compatible with the increase in the melting temperature as seen by DSC. A small increase in the interfacial roughness represents the overall rearrangement of the volume fillings.

Again, GM1 is seen to promote the interaction of Aβ monomers with the membrane, with a structural effect extending further than the surface layer. This occurrence is not captured by simulation studies, which found that the insertion of Aβ is restrained to the region of the GM1 headgroups [21], while it appears evident in the experiments in terms of internal agreement. This could suggest that deeper insertion requires longer times than the hundreds of nanoseconds explored by the simulation. Moreover, the simultaneous presence of cholesterol is not required for GM1 to perform a promoting role.

#### 2.3.2. Mixed PC-Phospholipid/Cholesterol Bilayers

In the previous section, we showed that some Aβ_mon_-membrane interaction, well developed in the PC:Chol:GM1 raft-mime membranes, also occurs in the presence of GM1 alone. Instead, in general, it is the presence of cholesterol to be claimed as crucial for rafts and for the organization of membrane domains in the liquid ordered (lo) arrangement [33], which displays unique structural features with functional correlates. In addition, it has been suggested that cholesterol is a risk factor for neurodegenerative diseases, but it is still controversial as to whether cholesterol and AD could have a mutual impact [34]. Cholesterol might promote the production of potentially toxic Aβ fragments [35]. Molecular dynamics studies have shown that cholesterol influences the ability of Aβ to embed in model membranes and to self-assemble into potentially toxic oligomeric species [36].

**Figure 4:**
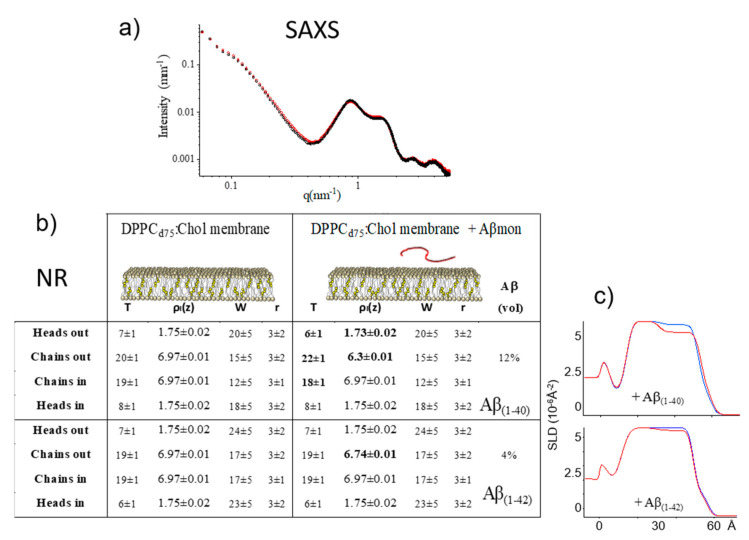
PC-phospholipid:cholesterol membranes exposed to Aβ monomers. (**a**) SAXS results. SAXS spectra of DPPC:Chol vesicles before (black diamonds) and after 1 h incubation with Aβ_mon(1–42)_ (red squares) T = 26 °C. Incubation with peptide was done for 1 h at T = 50 °C. NR results. (**b**) Table. Layers parameters, as deduced by the fits of NR spectra, of a single deposited DPPC_d75_:Chol membrane (10:1.25 mol/mol fraction), before (left section) and after (right section) interaction with Aβ_mon(1–40)_ and Aβ_mon(1–42)_. Layers thickness (Å), SLD (10^−6^ Å^−2^), solvent penetration (% in volume), roughness of interfaces (Å). (**c**) SLD profiles obtained by the best NR data fit of the DPPC_d75_:Chol (10:1.25 mol/mol) membranes before (blue) and after (red) interaction with Aβ_mon(1–40)_ (upper panel) and Aβ_mon(1–42)_ (lower panel). T = 22 °C. NR spectra are reported in the Figure S9. After incubation, full characterization of the membranes in three solvents was performed.

Thus, to elucidate whether cholesterol plays a role, independent of the presence of GM1, in promoting the insertion of nascent Aβ into model membranes, we performed both SAXS and NR experiments on DPPC:Chol model membranes at room temperature. The results are reported in Figure 4. Both techniques show that the DPPC:Chol membrane is insensitive to Aβ_mon_ addition. The SAXS spectra are nearly superimposable. Similar results were obtained at T = 50 °C (Figure S8). Very small changes were also observed by NR on the supported PC:Chol membranes, prepared according to the vesicle-fusion method, with optimal surface coverage, upon incubation with a micromolar solution with a peptide:lipid mole ratio as high as 1:3.

Then, the presence of cholesterol alone, with a Chol:PC molar ratio of 0.12, was not enough to improve the low accessibility of the phospholipid surface for Aβ_mon_ and/or to adapt the lipid core to host it, whether the molecular species was Aβ_(1–40)_ or Aβ_(1–42)_. The Aβ_(1–42)_ species, in particular, is seen to be excluded from membrane permeation. Conversely, the presence of GM1 alone allowed Aβ_mon_ to bind to the membrane surface and to penetrate the lipid core even at a low temperature (see the Table in Figure 3).

#### 2.3.3. Mixed PC-Phospholipid/Cholesterol/GM1 (Raft-Mime) Bilayers: From Intra-Membrane Restructuring to Inter-Membrane Correlation

Comparing the NR results of the cholesterol-free DPPC:GM1 and the raft-mime DPPC:Chol:GM1 membranes (see the table in Figure 3), we observe that, while the total amount of the peptide penetration is very similar, a different distribution of Aβ in the various layers of the target membrane occurs according to whether or not GM1 is coupled to cholesterol. In the DPPC:GM1 membrane, the outer leaflet, exposed to the approaching Aβ_mon_, is more affected than the inner leaflet, resulting in a cone-shaped insertion profile. A similar cone-shaped profile is also deduced in the floating membrane configuration (Figure S10 and Table S2). In contrast, in the raft-mime, Aβ monomers affect the membrane with a jug-shaped profile, seemingly the front of a forming pore, as sketched in Reference [28]. These different profiles suggest that GM1 and cholesterol play a synergistic role in shaping the penetration of Aβ monomers into the membrane. We already found that structural coupling occurs between GM1 and cholesterol in a raft-mime situation [37], the latter being preferentially distributed in the inner leaflet in the presence of GM1 on the outer leaflet. Moreover, a study on lipid monolayers suggested that cholesterol facilitates the binding of Aβ peptide to the ganglioside headgroups [38]. Notably, the raft-mime membrane is more accessible to nascent Aβ than the less complex membranes.

**Figure 5:**
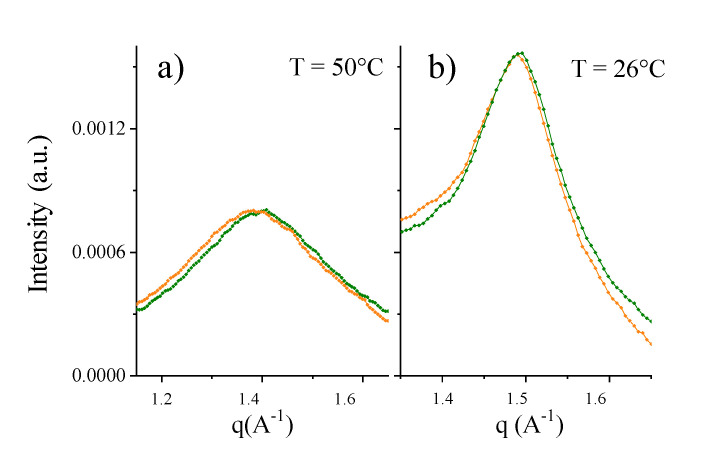
Raft-mime membranes upon interaction with Aβ monomers. Wide angle x-ray scattering (WAXS) of raft-mime asymmetric DPPC:Chol:GM1 = 10:1.25:0.5 bilayer before (olive curves) and after (orange curves) 1 h incubation of Aβ_(1–42)_ monomers at (0.2% mole fraction to the lipid). (**a**) T = 50 °C, in the fluid phase. (**b**) T = 26 °C, in the gel phase. In both phases, the local-arrangement-peaks shifted to lower q-values: from 1.396 Å^−1^ to 1.380 Å^−1^ at T = 50 °C and from 1.493 Å^−1^ to 1.490 Å^−1^ at T = 26 °C.

The results of the WAXS experiments on the raft-mime vesicles in solution reinforced this notion (Figure 5). In less complex paradigmatic systems (pure-PC, PC:Chol, and PC:GM1), the local arrangement (fluid and gel phase) was not altered; only the melting temperature was affected, indicating the influence of Aβ monomers on the propensity to assume a given state. Conversely, in the raft-mime system, the local packing of molecules in the core was altered. Because the fraction of Aβ is very low, this effect is notable and indicates a different overall lateral organization of the lipids on the few-Ångstrom-length scale, with an increased area per lipid at the interface.

Moreover, our further results suggest that the mutual interference of the Aβ monomers and raft-mime membranes is not limited to membrane and amyloid reshaping. It can extend to the external space involving the adjacent membranes, Aβ peptides acting as mediators or modulators of the interaction between the facing lipid rafts. In fact, ganglioside-enriched microdomains belonging to the adjacent cells’ membranes have been hypothesized to be able to spatially correlate to each other [39], providing a direct interaction site between platforms. As an experimental support to this hypothesis, we previously reported on the existence of bunches within multilayer stacks of mixed PC-GM1 membranes, evidenced by a split in the lamellar repeat distance corresponding to an in-register organization of compositionally distinct domains [40].

**Figure 6:**
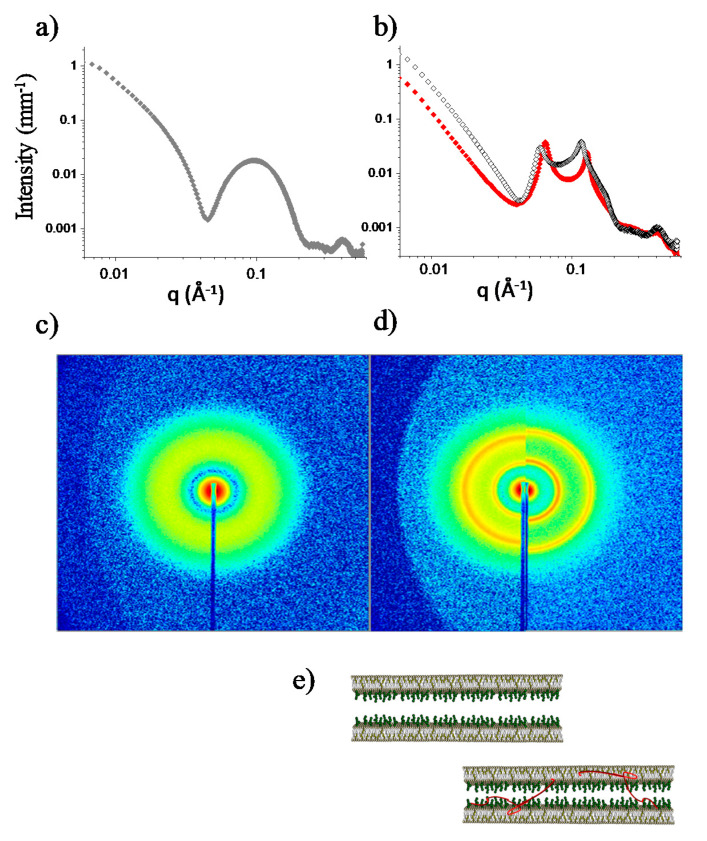
Aβ monomers intermediation between facing raft-mime bilayers. SAXS spectra and 2D images of raft-mime unilamellar vesicles (LUVs) reshaping upon osmotic stress. (**a**,**c**) before stress; (**b**,**d**) after stress with Aβ_mon_-free buffer (black open symbols in panel (**b**), and right image in panel (**d**)) and with buffer solution containing Aβ_mon(1–42)_ (0.2% mole fraction to the lipid) (red symbols in panel (**b**), left image in panel (**d**)). The rising of Bragg peaks, superimposed to the bilayer form factor, indicated the transition from unilamellar to multilayered vesicles. Peaks position was related to the characteristic inter-lamellar spacing of the raft-mime liposome in the absence and in the presence of Aβ_mon_. (**e**) Pictorial sketch of possible coordination between facing raft-mime membranes induced by nascent Aβ.

In the present study, we wondered whether Aβ monomers could interfere with this inter-membrane correlation. We tested this hypothesis by following the reorganization of raft-mime membranes after osmotic stress, an experimental protocol that allowed us to preserve the monomeric state and to approach the biomimetic concentration of Aβ. Gemini samples of the raft-mime LUVs in pure water were submitted to osmotic stress by adding either a buffered solution containing nascent Aβ_mon_ or the buffer alone. In both cases, the LUVs underwent a destabilization process [41], and they re-organized as multilayers (Figure 6), as shown by the rising of Bragg peaks superimposed to the bilayer form factor (Figure 6b). However, different reshaping occurred, corresponding to differences in the inter-lamellar spacing. The position of the peaks indicates that the characteristic inter-lamellar spacing of the raft-mime liposome in the absence of Aβ_mon_ is 107 Å (3-orders repeat at q_peak_ = 0.59, 0.118, and 0.177 Å^−1^); in the presence of Aβ_mon(1–42)_ (q_peak_ = 0.065, 0.130 Å^−1^), the spacing is 97 Å, 10 Å lower than the Aβ_mon(1–42)_-free case.

Surprisingly, the difference was not the one expected in the case of spacer molecules inserted in the interlamellar region, that is, forcing a larger distance between the lamellae in comparison to the Aβ-free case_._ Conversely, and notably, we observed that reshaping occurred with a definitely lower (by 10 Å) inter-lamellar distance. A reduction in the membrane thickness can be ruled out, as the spectra in the high-q region were nicely superimposed. While some dampening of bilayer fluctuations cannot be excluded, as a result of membrane dynamics perturbation due to peptide recruitment, it seems unlikely to be the sole reason for a 10 Å approach. Instead, a reduction in the thickness of the water interlayers could occur, corresponding to tightening the liposome shells upon coordination of the adjacent raft-mime membranes (as sketched in Figure 6e). Then, we suggest that nascent Aβ can coordinate the ganglioside headgroups belonging to the facing lamellae, thus behaving as mediators and modulators of the membrane-membrane interaction.

All the experimental results agreed in describing the structural interaction between nascent Aβ monomers and ganglioside-containing PC-membrane. At the membrane surface, interaction with nascent Aβ is allowed by the presence of gangliosides, but deep peculiar penetration is favored by the concomitant presence of cholesterol, asymmetrically disposed in the lipid core leaflets [37]. We can confirm that the lipid-raft platforms on plasma membranes constitute preferential loci for intimate recruitment of nascent Aβ at the early stage of formation, when in monomeric form.

## 3. Materials and Methods

1,2-dipalmitoyl-sn-glycero-3-phosphatidylcholine DPPC (SAXS, DSC), deuterated d_75_-DPPC (SANS), deuterated d_62_-DPPC (NR), and deuterated 1,2-dimyristoyl-sn-glycero-3-phosphatidylcholine d_67_-DMPC (SANS, DSC) were obtained from Avanti Polar Lipids (Alabaster, AL). Cholesterol was purchased from Sigma-Aldrich (Milano, Italy), and GM1 (Neu5Acα2-3(Galβ1-3GalNAcβ1-4)Galβ1-4Glcβ1Cer) was extracted and purified following the protocol of Tettamanti et al. [42].

Synthetic Aβ1-40 (DAEFRHDSGYEVHHQKLVFFAEDVGSNKGAIIGLMVGGVV) and Aβ1-42 (DAEFRHDSGYEVHHQKLVFFAEDVGSNKGAIIGLMVGGVVIA) peptides were prepared on a Syro I synthesizer (Biotage, Uppsala, Sweden) using the depsipeptide method [22]. The depsipeptide method is suitable for Aβ because it allows for avoiding the presence of seeds, preformed aggregates, or highly-folded structures in the starting solution, then preserving the starting pure monomer condition. Aliquots from the same batch of depsi-Aβ were stored in acidic solution (TFA 0.02%) at a concentration of 200 µM, and the native sequence was obtained by the switching procedure at basic pH immediately prior to each experiment. The peptide solutions were diluted to the final concentration (20–25 µM) in 50 mM of phosphate buffer (PB) pH 7.4 and used immediately.

Closed model membranes were prepared by dissolution of the components in the proper organic solvent, then mixing if required. After complete solvent removal in a rotating evaporator and under vacuum, the obtained lipid thin film was humidified and rehydrated (c = 15–20 mg/mL). The so-obtained lipids solutions were submitted to a freeze-and-thaw protocol, and then, if necessary, to extrusion, 71 passes through 800 Å pores, to obtain LUVs.

Langmuir-Blodgett and Langmuir-Schaefer techniques [43] were used to deposit the supported membranes onto single crystals of silicon (5 × 5 × 1.5 cm^3^) polished on one large face (111) and cleaned before use in the appropriate organic solvents.

AFM: Aliquots of synthetic Aβ at a concentration of 25 μM were incubated for 0.5–2 min on mica disks. After washing and drying, images were acquired on a Nanoscope III Multimode AFM (Veeco/Digital Instruments, Santa Barbara, CA, USA) operating in tapping mode (1 Hz scan speed).

Laser light scattering: Both static and dynamic laser light scattering measurements were carried out on the Aβ solutions at a concentration of 25 μM with a homemade apparatus, which includes a diode laser (λ = 532 nm), a temperature-controlled cell (T = 25 °C), and a digital correlator (Brookhaven Instruments Co, Holtsville, NY, USA).

SANS: The SANS experiments were performed on the D33 diffractometer at the ILL (Grenoble, France; experiment: doi:10.5291/ILL-DATA.8-03-893) and on the PACE spectrometer at the LLB (Saclay, France). Toward that end, 2 mm thick quartz cells (Hellma GmbH & Co. KG, Müllheim, DE, USA) were used to load with the samples. Scattered intensity was measured in the q-range 3 × 10^−3^ ≤ *q* ≤ 0.3 Å^−1^. Measured intensities were calibrated to absolute values (d∑/dΩ, mm^−1^) using normalization by the attenuated direct beam classical method. Standard procedures for absolute intensity calibration and for data correction for transmission, detector efficiency, and backgrounds were applied with a homemade program at PACE (PASiNET. MAT) [44]. Data analysis was performed using SasView 4.2.1 software [45].

The shape of the scattering curves was modeled using the form factor *P*(*q*) of polydisperse multishell spherical particles (*i* = 4), assuming the same SLD for the core (*i* = 1) and the solvent as for closed bilayers/vesicles. The three concentric shells corresponded to the lipid core of the membrane enclosed between two headgroup regions:

$$P\left(q\right)÷{\left[\overset{4}{\underset{i=1}{\sum }}3V_{i}\left(\rho_{i}-\rho_{\mathrm{i+1}}\right)\frac{\sin\left(qR_{i}\right)-qR_{i}\cos\left(qR_{i}\right)}{{\left(qR_{i}\right)}^{3}}\right]}^{2}$$

where *V_i_* and *R_i_* indicate the volume and radius of the concentric spheres, and *ρ_i_* are the SLDs of the core (*i* = 1), internal headgroups (*i* = 2), lipid chains (*i* = 3), and external headgroups (*i* = 4). *ρ*_5_ is the scattering length density of the solvent. The polydispersity of the particle size was determined based on Schultz distribution; the polidispersities of the shell thicknesses were determined based on Gaussian distribution. The beam linewidth was ∆λ/λ = 5%. The SLD of the solvent was set at the value of pure D_2_O (6.36 × 10^−6^ Å^−2^) in the case of phospholipid vesicle solutions and at the value of mixed D_2_O:H_2_O 10:1 (5.65 × 10^−6^ Å^−2^) after injection of the Aβ peptide solutions.

X-ray scattering (SAXS, WAXS): The SAXS and WAXS experiments were conducted on the high-brilliance ID02 beamline at ESRF (Grenoble, France) in the region of momentum transfer 0.002 ≤ *q* ≤ 3 Å^−1^. Samples were loaded in plastic capillaries (KI-beam, ENKI, Italy) and put onto a temperature-controlled multi-place sample holder, allowing for nearly simultaneous measurements of the sample and reference cells. Several short frames (0.1 s) were acquired and summed up, after comparison, to avoid any radiation damage. After data normalization and correction, the cell and solvent contributions were subtracted from each spectrum.

DSC: The DSC experiments were performed with a modulated adiabatic scanning calorimeter (MASC) instrument [46], which measures the difference in the power absorbed or released by both the sample solution and its buffer during a temperature scan, with respect to a reference (water). Instrumental drifts were then cancelled by this double difference. All the samples (0.2 mL) were put in sealed glass capillaries and submitted to subsequent temperature cycles in the temperature range of 33 °C < T < 51 °C at a scan rate of 0.25 °C/min, in heating and cooling modes.

NR: The NR experiments were performed on the FIGARO beamline at ILL (Grenoble, France; experiment: doi:10.5291/ILL-DATA.8-02-733). Reflectivity was measured in time-of-flight (TOF) mode at two incident angles incident angles θ_1_ = 0.8° and θ_2_ = 3.2° at the silicon-water interface, with neutrons coming from the silicon side. Reflectivity from the bare silicon support and from each model membrane before and after peptide interaction was measured in different contrast solutions. Contemporary fits of the data have been carried out with Motofit software [47], modeling the membranes as a sequence of four layers, that is, the hydrophilic and hydrophobic portion of each membrane leaflet. The data fit provides information on the thickness, SLD, solvent penetration, and roughness of each layer, allowing for reconstructing the SLD profile of the investigated samples.

## 4. Conclusions

We investigated the effect of nascent Aβ on the organization of cell-membrane models, mimicking its fate immediately after its release from the APP. In fact, the cell membrane is likely to be the first interaction site of nascent Aβ due to proximity and simple diffusion.

Our results indicate that the membrane properties are the primary determinants of the interaction with the Aβ monomers. In contrast, the specific configuration of the Aβ monomers does not seem to play a significant role at this stage, supporting the idea that the N-terminal region of nascent Aβ is responsible for the interaction with the cell membrane.

Aβ monomers are able to interact with fluid phospholipid bilayers. In raft-mime conditions, the interaction is boosted by the simultaneous presence of cholesterol and the GM1-ganglioside. Nascent Aβ monomers promote entanglement between raft-mime membranes, a basic structural process that might be involved in cell-cell adhesion and cell motility [48].

We speculate that the role of nascent Aβ monomers, autonomous and aside from the amyloidogenic route, may be double-edged. They are generated in the setting of a rescue and adaptive response, but due to their propensity to interact with and entangle membranes, they may inevitably cause the disruption of brain cell membrane architecture over time, thereby playing an intrinsic part in the aging of the underlying natural brain. In this latter role, Aβ monomers may contribute to altering the composition and distribution of plasma membrane lipids by preferential interactions [49] and to reducing both brain plasticity and the cell-to-cell communications afforded by extracellular vesicles [50]. In addition, the change in membrane fluidity and composition, including the modification of the ganglioside pattern following aging or disease [51,52,53], might impact the physiological or pathological fate of Aβ monomers. Consequently, Aβ monomers might represent relevant and novel targets of pharmacological intervention from both therapeutic and preventive perspectives.

The present work provides structural grounding for novel perspectives, and it advances the current understanding of the structural events that might be associated with the brain repair function and the pathological and physiological brain decline.

## Supplementary Materials

Supplementary materials can be found at https://www.mdpi.com/1422-0067/21/21/8295/s1. Figure S1: Presence of un-aggregated Aβ monomers. Figure S2: Evaluation of the degree of monomeric state for Aβ. Figure S3: PC-phospholipid bilayers upon interaction with Aβmon (SANS). Figure S4: PC-phospholipid bilayers upon solvent addition (SANS). Table S1: Structural parameters of PC vesicles. Figure S5: Spectra of DPPC vesicles T = 25 °C. Figure S6: SAXS spectra of DPPC vesicles. Figure S7: Aβ_mon_ interaction with DMPC (DSC). Figure S8: SAXS spectra of DPPC:Chol vesicles. Figure S9. Aβ_mon_ interaction with DPPC:Chol membranes (NR). Figure S10: Aβ_mon_ interaction with DPPC:GM1 floating membrane (NR). Table S2: Layers parameters, as deduced by the fits of NR spectra.
