# Supplementary material for: Aβ Beyond the AD Pathology: Exploring the Structural Response of Membranes Exposed to Nascent Aβ Peptide
Source: Int J Mol Sci. 2020 Nov 5;21(21):8295. doi: 10.3390/ijms21218295 (PMC7663943; doi:10.3390/ijms21218295)
Supplement: Supplementary file 1 [file ijms-21-08295-s001.pdf]

Supplemental Information

Aβ Beyond the AD Pathology: Exploring the Structural Response of Membranes Exposed to Nascent Aβ Peptide

Valeria Rondelli[1], Mario Salmona[2*], Laura Colombo[2], Giulia Fadda[4], Giovanna Fragneto[3], Laura Cantu' [1*], Elena Del Favero[1]

[1] Dept. Medical Biotechnologies and Traslational Medicine, Università of Milano, Via F.lli Cervi, 93, 20090 Segrate (MI), Italy
[2] Dept. of Molecular Biochemistry and Pharmacology, Istituto di Ricerche Farmacologiche Mario Negri IRCCS, Via Mario Negri, 2, 20156 Milano, Italy
[3] Institut Laue-Langevin, 71 Avenue des Martyrs, BP 156, 38000 Grenoble Cedex, France
[4] Université Paris 13, UFR SMBH, 74 rue Marcel Cauchin, 93017 Bobigny, France, Laboratoire Leon Brillouin, CEA Saclay, F-91191 Gif sur Yvette Cedex, France

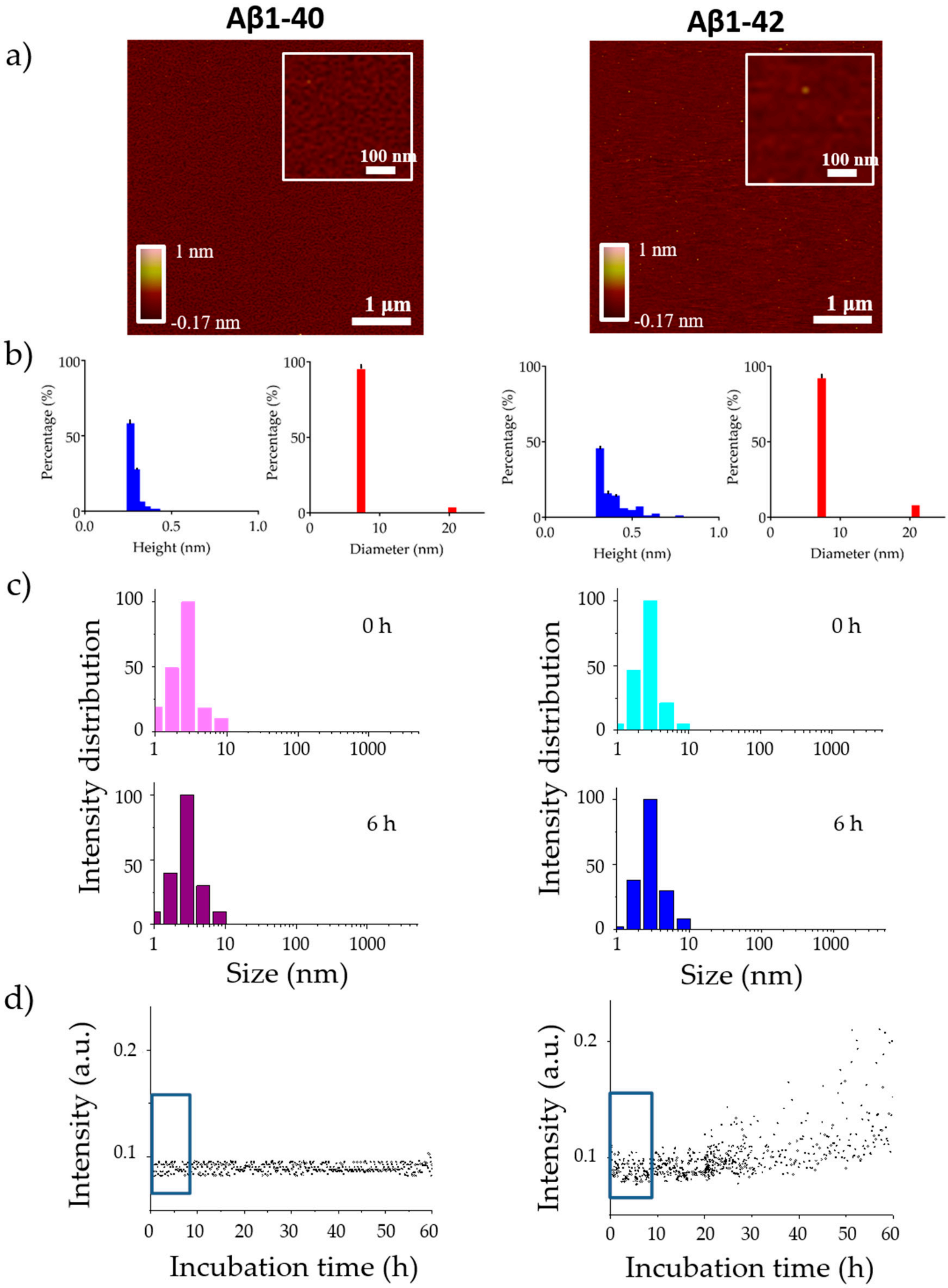

Figure S1. **Presence of un-aggregated Aβ monomers.** Top: a) Representative taping mode AFM images (height data) and b) corresponding size histograms of the $A\beta_{1\text{-}40}$ and $A\beta_{1\text{-}42}$ particulate present in a 25 μM solution in phosphate buffer (PB) 50 mM, pH 7.4, just after dissolution. Bottom: c) size distribution obtained by dynamic laser light scattering measurements on $A\beta_{1\text{-}40}$ and $A\beta_{1\text{-}42}$ 25 μM solutions in PB 50 mM, pH 7.4, T = 25 °C just after dissolution and at 6 h from dissolution; d) time evolution of the scattered laser light intensity for $A\beta_{1\text{-}40}$ and $A\beta_{1\text{-}42}$ 25 μM solutions in PB 50 mM, pH 7.4, T = 25 °C. The time-interval used for scattering experiments on membranes (1-6 h) is marked.

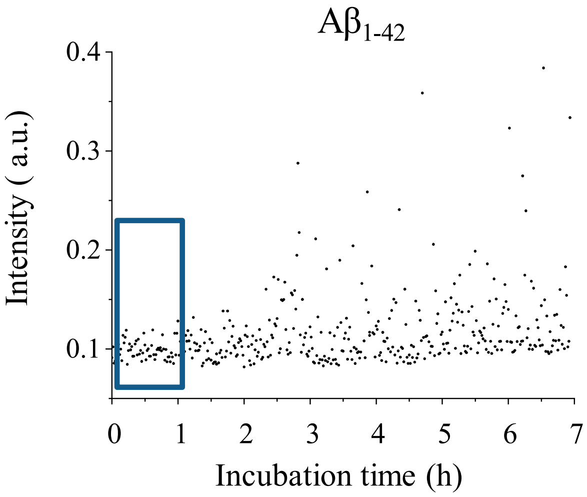

Figure S2. Evaluation of the degree of monomeric state for $A\beta_{1\text{-}42}$ 25 μM at T 50 °C to assess the feasibility of SAXS and WAXS experiments. We assessed that 1 h delay (marked box) is largely within requirements by measuring the time evolution of the scattered laser light intensity in PB 50 mM, pH 7.4.

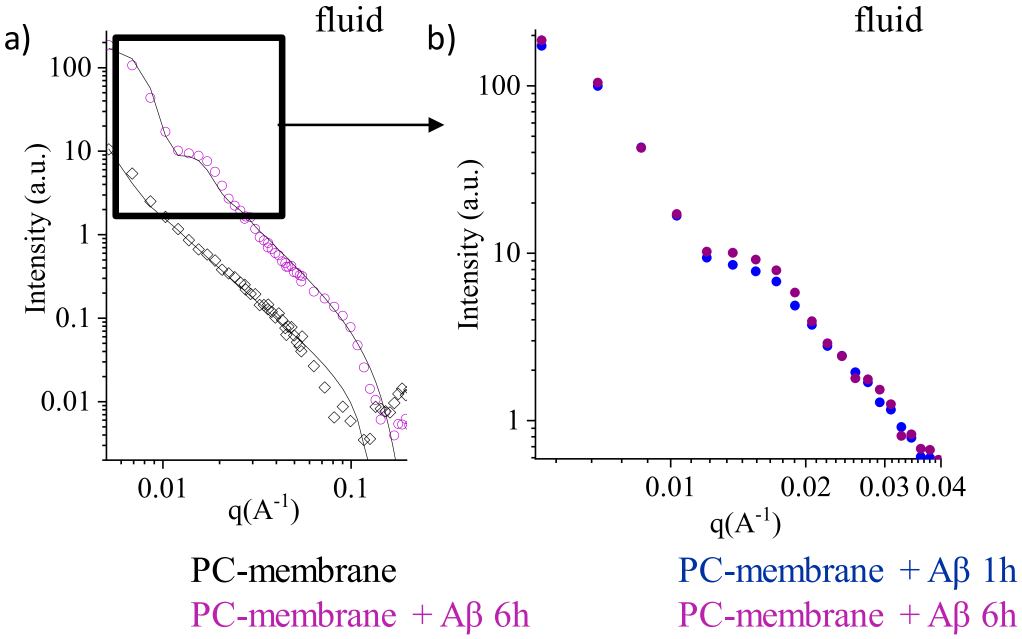

Figure S3. PC-phospholipid bilayers upon interaction with Aβmon (SANS). $A\beta_{mon(1\text{-}42)}$ interaction with dimyristoyl phosphatidylcholine (DMPC) unilamellar vesicles. T = 25°C, above the chain melting temperature, in the fluid-phase. a) Small angle neutron scattering (SANS) spectra of $DMPC_{d67}$ unilamellar vesicles in D_2O (c = 20 mg/ml) before (black open diamonds) and after 6h of incubation with $A\beta_{mon(1\text{-}42)}$ at 0.2% mole ratio (violet open dots), 20 μM in solution. Lines are the fitting curves with the form factor reported in the Materials and Methods. Structural parameters are reported in **Table S1**. b) Focus on the restricted area enclosed in the rectangle of panel a) (violet dots) added with the spectrum of the same sample within the first hour of incubation. The progressive change induced on phospholipid membranes can be seen.

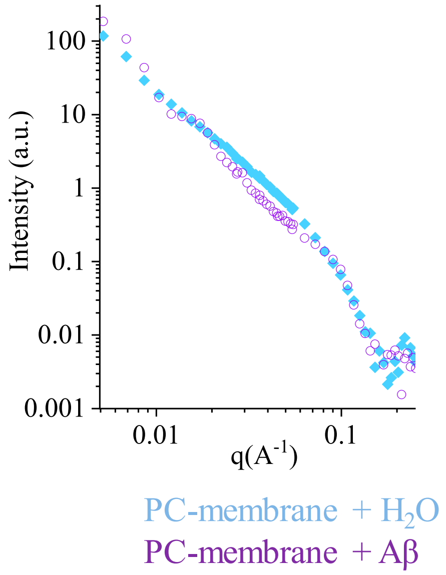

Figure S4. Comparison between the spectra of DMPC unilamellar vesicles in 250 µl D_2O (c = 20 mg/ml) after 6h incubation with 20 µl H_2O (cyan diamonds) or with $A\beta_{mon(1-42)}$ (dissolved in 20 µl H_2O).

Table S1. Structural parameters of PC vesicles. Structural parameters of $DPPC_{d75}$ unilamellar vesicles (c = 15 mg/ml) before and after 6h of incubation with $A\beta_{mon}$ (1-40) at 0.2% mole ratio, T = 50°C, and of $DMPC_{d67}$ unilamellar vesicles (c = 20 mg/ml) before and after 6h of incubation with $A\beta_{mon}$ (1-42) at 0.2% mole ratio (violet open dots), T = 25°C. Vesicle Radius (Å) and (polidispersity); Layers thickness T (Å), scattering length density ϱ (10^{-6} Å^{-2}). The values of the scattering length density of the outer surface layer of the vesicles are evidenced in ***italics***.

		**$DPPC_{d75}$**		**$DMPC_{d67}$**	
**membrane**	**Radius**	310	(0.28)	290	(0.35)
		**T**	**ϱ**	**T**	**ϱ**
	**Heads out**	5	***5.5***	5	***5***
	**Chains**	37	7.55	35	7.6
	**Heads in**	5	5.5	5	5
**membrane + $A\beta_{mon}$**	**Radius**	310	(0.23)	290	(0.2)
		**T**	**ϱ**	**T**	**ϱ**
	**Heads out**	7	***4.3***	7	***4***
	**Chains**	36	7.55	35	7.6
	**Heads in**	5	5.5	5	5

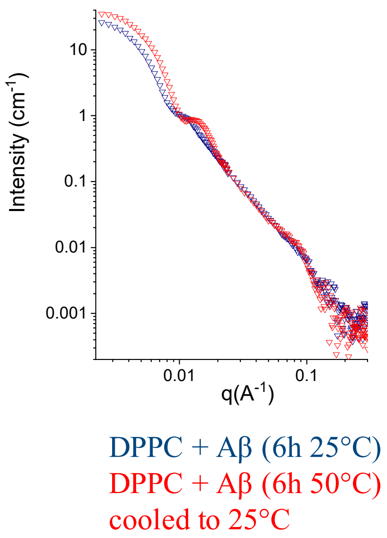

Figure S5. T = 25°C. Spectra of DPPC$_{d75}$ unilamellar vesicles in D_2O after 6h of incubation with Aβ$_{mon(1-40)}$ performed at T = 25 °C (blue triangles) and T = 50 °C (red triangles). Differences are visible in the low-q region of the spectra.

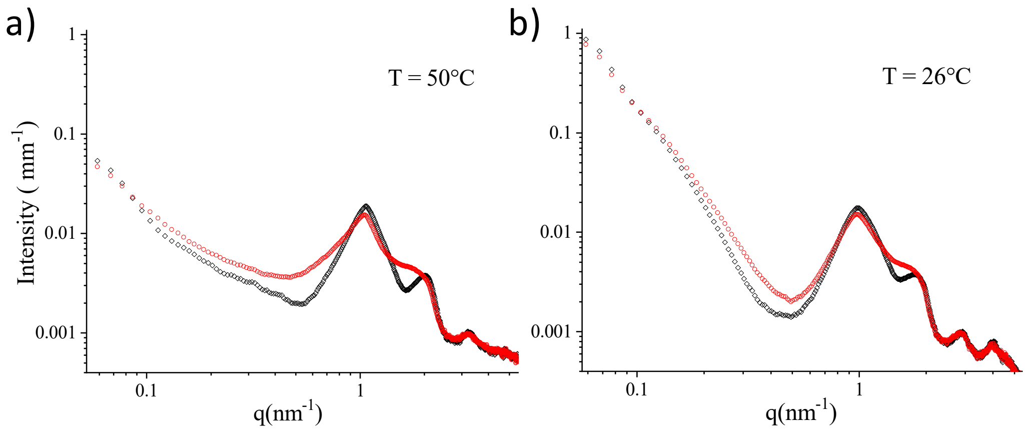

Figure S6. Small angle X-ray scattering (SAXS) spectra of DPPC vesicles (black diamonds) and (DPPC + Aβ$_{mon(1-42)}$) vesicles (red dots). a) T = 50 °C in the fluid phase, b) T = 26°C (right panel) in the gel phase. Although the presence of peaks around q = 1 nm^{-1} shows that vesicles are not perfectly unilamellar and spherical, some effect of Aβ$_{mon(1-42)}$ on the intensity profile is visible at q < 2 nm^{-1}.

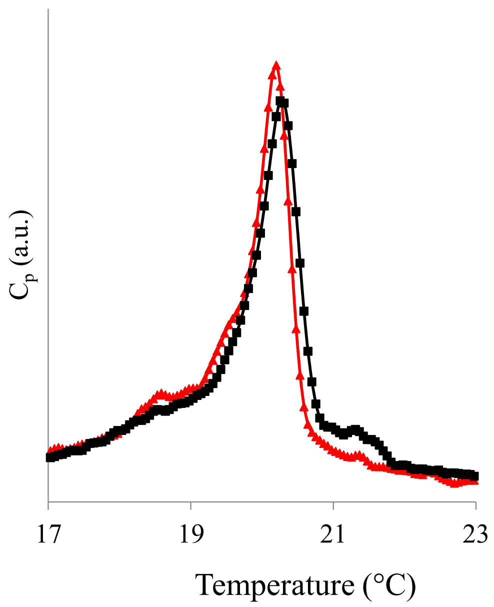

Figure S7. Aβ$_{mon}$ interaction with DMPC (DSC). Upward T-scan of DMPC$_{d67}$ vesicles (black) and DMPC $_{d67}$ + Aβ$_{mon}$(1-40) vesicles (red). c = 20 mg/ml, scan rate 0.25°C/min. The interaction with Aβ$_{mon(1-40)}$ at a peptide:lipid = 0.2% mole ratio, induced a ~ 0.1°C downshift of the transition temperature (main peak from 20.30 to 20.18°C).

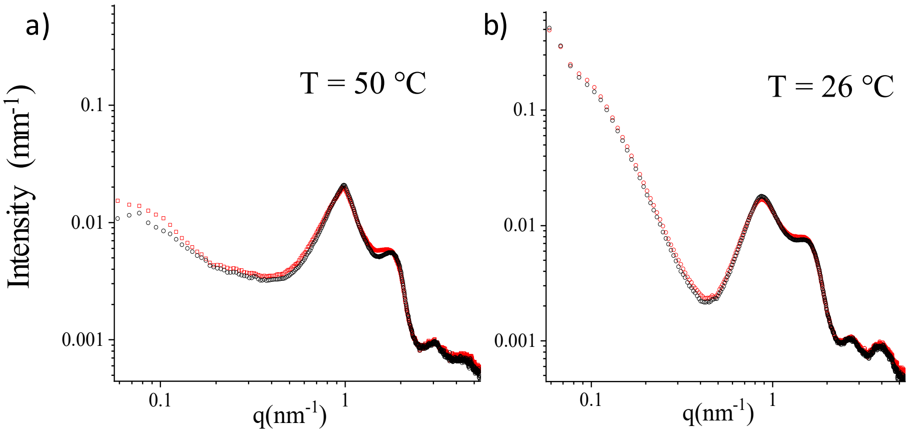

Figure S8. Small angle X-ray scattering (SAXS) spectra of DPPC:Chol vesicles (black diamonds) and (DPPC:Chol + Aβ$_{mon(1-42)}$) vesicles (red dots). a) T = 50 °C in the fluid phase, b) T = 26°C (right panel) in the gel phase. Although the presence of peaks around q = 1 nm^{-1} shows that vesicles are not perfectly unilamellar and spherical, it can be appreciated that the spectra are nearly unchanged after incubation of Aβ$_{mon(1-42).}$

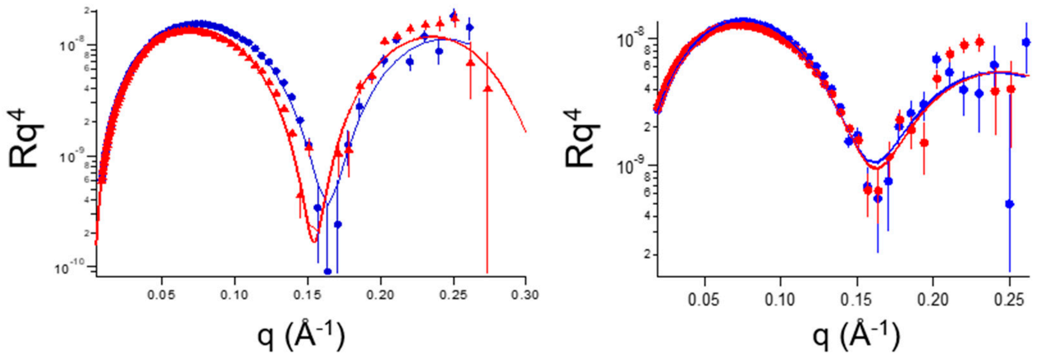

Figure S9. Aβ$_{mon}$ interaction with DPPC:Chol membranes (NR). NR spectra of the DPPC$_{d62}$:Chol membranes before (blue) and after (red) interaction with Aβ$_{mon(1-40)}$ (left panel) and Aβ$_{mon(1-42)}$ (right panel).

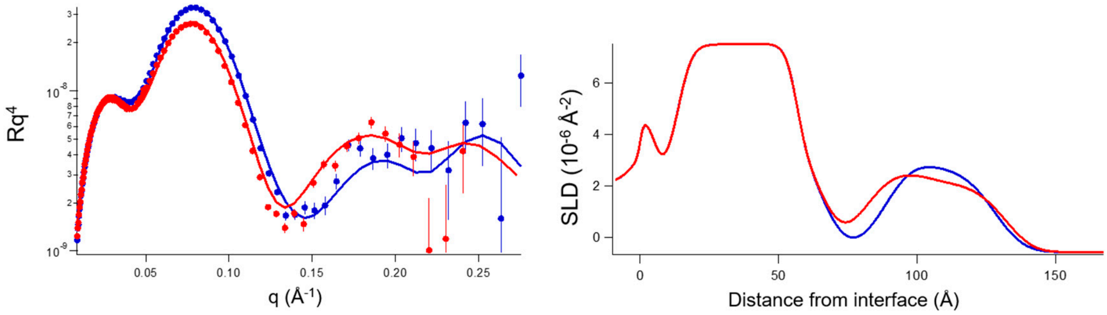

Figure S10. Aβ$_{mon}$ interaction with DPPC:GM1 floating membrane (NR). NR results in the floating-membrane configuration. Spectra and SLD profiles obtained by the best NR data fit of the DPPC$_{d75}$ + GM1 floating membrane before (blue) and after (red) interaction with Aβ$_{mon(1-40)}$. The supporting membrane is DSPC.

Table S2. Layers parameters, as deduced by the fits of NR spectra of Figure S7. The top block describes the DPPC$_{d75}$ + GM1 floating membrane (5% total GM1 molar ratio to lipids, asymmetrically residing only in the external leaflet), the bottom block describes the DSPC supporting membrane.

Layers thickness (Å), scattering length density (10^{-6} Å^{-2}), solvent penetration (% in volume), roughness of interfaces (Å). In the forelast column, the estimated peptide penetration at different depths within the membrane.

		Floating DPPC-GM1					Floating DPPC-GM1 +wt 1-40				
		**thick**	**rho**	**solv p**	**rough**		**thick**	**rho**	**solv p**	**rough**	**abeta**
**heads out**		7	4.21	65	7		8	3.6	65	8	22%
**chains out**		16	7.16	60	7		22	6	60	8	20%
**chains in**		16	7.91	59	7		19	7.14	59	8	12%
**heads in**		7	4.98	65	7		7	4.52	65	8	13%
**solv**		20			7		13			7	
**heads out**		8	4.98	15	4		8	4.98	15	4	
**chains out**		19	7.96	5	7		19	7.96	5	7	
**chains in**		23	7.96	5	4		23	7.96	5	4	
**heads in**		9	4.21	15	7		9	4.21	15	7	
